# Supplementary material for: SARS-CoV-2 vaccine breakthrough infections with the alpha variant are asymptomatic or mildly symptomatic among health care workers
Source: Nat Commun. 2021 Oct 15;12:6032. doi: 10.1038/s41467-021-26154-6 (PMC8521593; doi:10.1038/s41467-021-26154-6)
Supplement: Supplementary file 1 — Description of Additional Supplementary Files [file 41467_2021_26154_MOESM1_ESM.pdf]

**Description of Additional Supplementary Files**

File Name: Supplementary Data 1
Description: The anonymized data relevant to SARS-CoV-2 infection during the study period in vaccinated and control subjects, along with serologic results indicating their previous exposure to SARS-CoV-2 infection, are reported.
