# Supplementary material for: SARS-CoV-2 vaccine breakthrough infections with the alpha variant are asymptomatic or mildly symptomatic among health care workers
Source: Nat Commun. 2021 Oct 15;12:6032. doi: 10.1038/s41467-021-26154-6 (PMC8521593; doi:10.1038/s41467-021-26154-6)
Supplement: Supplementary file 3 — Reporting Summary [file 41467_2021_26154_MOESM3_ESM.pdf]

nature research

Corresponding author(s): Daniele Lilleri

Last updated by author(s): Sep 2, 2021

Reporting Summary

Nature Research wishes to improve the reproducibility of the work that we publish. This form provides structure for consistency and transparency in reporting. For further information on Nature Research policies, see our Editorial Policies and the Editorial Policy Checklist.

Statistics

For all statistical analyses, confirm that the following items are present in the figure legend, table legend, main text, or Methods section.

n/a	Confirmed	
☐	☒	The exact sample size (*n*) for each experimental group/condition, given as a discrete number and unit of measurement
☒	☐	A statement on whether measurements were taken from distinct samples or whether the same sample was measured repeatedly
☐	☒	The statistical test(s) used AND whether they are one- or two-sided<br>*Only common tests should be described solely by name; describe more complex techniques in the Methods section.*
☒	☐	A description of all covariates tested
☒	☐	A description of any assumptions or corrections, such as tests of normality and adjustment for multiple comparisons
☐	☒	A full description of the statistical parameters including central tendency (e.g. means) or other basic estimates (e.g. regression coefficient) AND variation (e.g. standard deviation) or associated estimates of uncertainty (e.g. confidence intervals)
☐	☒	For null hypothesis testing, the test statistic (e.g. *F*, *t*, *r*) with confidence intervals, effect sizes, degrees of freedom and *P* value noted<br>*Give P values as exact values whenever suitable.*
☒	☐	For Bayesian analysis, information on the choice of priors and Markov chain Monte Carlo settings
☒	☐	For hierarchical and complex designs, identification of the appropriate level for tests and full reporting of outcomes
☒	☐	Estimates of effect sizes (e.g. Cohen's *d*, Pearson's *r*), indicating how they were calculated

Our web collection on statistics for biologists contains articles on many of the points above.

Software and code

Policy information about availability of computer code

Data collection	No software was used
Data analysis	SARS-CoV-2 lineages were assigned from alignment file using the Phylogenetic Assignment of Named Global Outbreak LINeages tool PANGOLIN v1.07 (https://github.com/hCoV-2019/pangolin).

For manuscripts utilizing custom algorithms or software that are central to the research but not yet described in published literature, software must be made available to editors and reviewers. We strongly encourage code deposition in a community repository (e.g. GitHub). See the Nature Research guidelines for submitting code & software for further information.

Data

Policy information about availability of data

All manuscripts must include a data availability statement. This statement should provide the following information, where applicable:

- Accession codes, unique identifiers, or web links for publicly available datasets
- A list of figures that have associated raw data
- A description of any restrictions on data availability

The anonymized data relevant to SARS-CoV-2 infection during the study period in vaccinated and control subjects, along with serologic results indicating their previous exposure to SARS-CoV-2 infection, are available in the Dryad database under DOI 10.5061/dryad.n2z34tmxk [https://doi.org/10.5061/dryad.n2z34tmxk]. Raw data associated to Figures 2a-c are present in the dataset .The 23 SARS-CoV-2 sequences obtained in this study are openly available on GISAID portal and European Nucleotide Archive under the accession numbers EPI_ISL_3836237-EPI_ISL_3836259.

Field-specific reporting

Please select the one below that is the best fit for your research. If you are not sure, read the appropriate sections before making your selection.

- [x] Life sciences
- [ ] Behavioural & social sciences
- [ ] Ecological, evolutionary & environmental sciences

For a reference copy of the document with all sections, see nature.com/documents/nr-reporting-summary-flat.pdf

Life sciences study design

All studies must disclose on these points even when the disclosure is negative.

Sample size	No sample size calculation was required. We enrolled in the study all healthcare workers of Fondazione IRCCS Policlinico San Matteo who received vaccination (n=3720) and a control group of healthcare workers (n=346) who did not receive vaccination during the study period. This sample sizes were sufficient to verify immune protection from secondary infections conferred by natural SARS-CoV-2 infection in the same population (Rovida et al., Int J Infect Dis 2021; 109:199-202).
Data exclusions	No data were excluded from the analysis.
Replication	Samples were tested once because the amount of blood collected did not allow repeated measures.
Randomization	This was an observational study, which did not involve any intervention beyond the standard of care ongoing during the study period. No randomization was required by the study. Subjects who received vaccination were enrolled in the vaccine group, while subjects who did not receive vaccination during the study period were enrolled in the control group. Both vaccinated and control group were exposed to the same risk of SARS-CoV-2 infection, since all of them were healthcare workers of the same Institution. The decision to defer vaccination was taken by the individual subjects.
Blinding	Blinding was not applicable, because this was an observational non-randomized study. The vaccine efficacy was already investigated in a randomized controlled study. The aim of the present study was instead to investigate vaccine effectiveness in real-life observational study.

Reporting for specific materials, systems and methods

We require information from authors about some types of materials, experimental systems and methods used in many studies. Here, indicate whether each material, system or method listed is relevant to your study. If you are not sure if a list item applies to your research, read the appropriate section before selecting a response.

Materials & experimental systems

n/a	Involved in the study	
x		Antibodies
	x	Eukaryotic cell lines
x		Palaeontology and archaeology
x		Animals and other organisms
	x	Human research participants
x		Clinical data
x		Dual use research of concern

Methods

n/a	Involved in the study	
x		ChIP-seq
x		Flow cytometry
x		MRI-based neuroimaging

Eukaryotic cell lines

Policy information about cell lines

Cell line source(s)	Vero C1008 (Vero 76, clone E6, Vero E6; ATCC1CRL-1586TM)
Authentication	The cell line used was not authenticated
Mycoplasma contamination	Cells were not tested for Mycoplasma contamination
Commonly misidentified lines (See ICLAC register)	No commonly misidentified cell lines were used in the study.

Human research participants

Policy information about studies involving human research participants

Population characteristics	Healthcare workers were enrolled; median age: 47, range 21-70 years; sex: 58.5% female, 31.5% male.
Recruitment	Healthcare workers of Fondazione IRCCS Policlinico San Matteo, Pavia receiving BNT162b2 vaccine were recruited. All subjects who received complete vaccination within March 31 2021 were enrolled. A control group constituted by all the subjects who did not receive vaccination until the study end (May 10 2021) was also enrolled. Subjects who received a single vaccine dose during the study period were not enrolled.
Ethics oversight	Comitato Etico Area Pavia.

Note that full information on the approval of the study protocol must also be provided in the manuscript.
